# Intra- and inter-observer variability in measurement of target lesions: implication on response evaluation according to RECIST 1.1

**DOI:** 10.2478/v10019-012-0009-z

**Published:** 2012-01-02

**Authors:** Daniela Muenzel, Heinz-Peter Engels, Melanie Bruegel, Victoria Kehl, Ernst J. Rummeny, Stephan Metz

**Affiliations:** 1 Department of Radiology, Technische Universität München; Munich, Germany; 2 Institute of Medical Statistics and Epidemiology, Technische Universität München; Munich; Germany; 3 Munich Study Center; Munich, Germany

**Keywords:** tumour measurement, RECIST, PACS, LMS

## Abstract

**Background:**

The assessment of cancer treatment in oncological clinical trials is usually based on serial measurements of tumours’ size according to the Response Evaluation Criteria in Solid Tumours (RECIST) guidelines. The aim of our study was to evaluate the variability of measurements of target lesions by readers as well as the impact on response evaluation, workflow and reporting.

**Patients and methods:**

Twenty oncologic patients were included to the study with CT examinations from thorax to pelvis performed at a 64 slices CT scanner. Four readers defined and measured the size of target lesions independently at baseline and follow-up with PACS (Picture Archiving and Communication System) and LMS (Lesion Management Solutions, Median technologies, Valbonne Sophia Antipolis, France), according to the RECIST 1.1 criteria. Variability in measurements using PACS or LMS software was established with the Bland and Altman approach. The inter- and intra-observer variabilities were calculated for identical lesions and the overall response per case was determined. In addition, time required for evaluation and reporting in each case was recorded.

**Results:**

For single lesions, the median intra-observer variability ranged from 4.9–9.6% (mean 5.9%) and the median inter-observer variability from 4.3–11.4% (mean 7.1%), respecting different evaluation time points, image systems and observers. Nevertheless, the variability in change of Δ sum longest diameter (LD), mandatory for classification of the overall response, was 24%. The overall response evaluation assessed by a single respectively different observer was discrepant in 6.3% respectively 12% of the cases compared with the mean results of multiple observers. The mean case evaluation time was 286s *vs*. 228s at baseline and 267s vs. 196s at follow-up for PACS and LMS, respectively.

**Conclusions:**

Uni-dimensional measurements of target lesions show low intra- and inter-observer variabilities, but the high variability in change of Δ sum LD shows the potential for misclassification of the overall response according to the RECIST 1.1 guidelines. Nevertheless, the reproducibility of RECIST reporting can be improved for the case assessment by a single observer and by mean results of multiple observers. Case-based evaluation time was shortened up to 27% using custom software.

## Introduction

The accurate assessment of tumour size is essential for clinical oncological trials.^1^ Decision on the subsequent cancer treatment often depends on radiological reports about current status and changes in tumour burden.^2^,^3^ For comparison and interpretation of oncological trial results it is important to classify measurements of tumour burden consistently and reproducible, independent of different clinical institutions and observers. Definite guidelines for standardization of tumour measurements and response evaluation were published in 2000 as a Response Evaluation Criteria in Solid Tumours (RECIST) criteria.^4^ These guidelines define the selection of target lesions in terms of number, localization, minimal tumour size and measurability. Parameters for the overall response evaluation are progressive disease (PD), stable disease (SD), partial response (PR) and complete remission (CR). Beside a high accuracy for the quantification of tumour progress or shrinkage it is desirable to simplify and shorten international guidelines as far as possible. In this context, the revised RECIST guidelines 1.1 were published in 2008 with, amongst others, changes in the total number of target lesions (5, formerly 10) and in standards for measurement of *e.g*. lymph nodes (1.5 cm short axis minimum for target lymph node).^5^ However, quantitative reporting in clinical routine with measurements of multiple lesions is costly and time-consuming, but would be desirable for each oncologic patient.

The aim of our study was to evaluate the variability of target lesion measurements by readers as well as the impact on overall response evaluation, workflow and reporting.

## Patients and methods

### Study population

Twenty oncologic patients (11 male, 9 female, mean age 60±14 years) were included, selected randomly from our clinical study archive. Primary tumour histology was lung cancer (NSCLC n=6, SCLC n=1), colon cancer (n=3) and urothelium cancer (n=3) as well as n=1 each for cancer of pancreas, breast cancer, endometrial cancer, teratoma, germ cell tumour, and lymphoma. All patients had two CT examinations from thorax to pelvis (at baseline and follow-up), performed at a 64 slices CT scanner (Siemens, Forchheim, Germany) with the application of intravenous contrast agent in all cases.

### Image analysis

Evaluation was performed on images with a reconstruction kernel of 30 and a slice thickness of 5 mm, but both, the soft tissue (window width, 500HU; window level, 55HU) and the lung window (window width, 1,500HU; window level, −600HU) setting could be applied. Uni-dimensional (1D) measurements of target lesions for baseline and follow-up were performed according to the RECIST 1.1 guidelines, non-target or new lesions were not respected. The target lesions were not preselected, thus each observer defined individually appropriate lesions. Note, target lesions defined at baseline and invisible in follow-up examinations were excluded from statistical computations.

Four radiologic specialists with more than 5 years experience in oncologic radiology performed in our study. At the end, each observer had prepared 4 reports per case, one each for baseline and follow-up for both, PACS and LMS. The lag time between readings was at least 4 weeks and case evaluation was prepared in a random order.

### PACS (Picture Archiving and Communication System)

Previous tumour measurements were not shown and actual measurements not stored within the images. Results of PACS-based assessments were documented using a standard, handwritten EORTC (European Organization for Research and Treatment of Cancer) formula. Patient and examination data as well as 1D-measurements for target lesions, slice position (z-orientation) and potential individual descriptive comments for clarification (*e.g*. liver metastasis, segment five) were listed. Anatomic subsumption was set according to the following categorization: 1 = primary tumour; 2 = lymph node; 3 = lung metastasis; 4 = liver metastasis; 7 = skin metastasis; 8 = other soft tissue metastasis; 9 = other metastasis. The sum of the longest diameters (LD) of the target lesions per case was calculated for baseline and follow-up examinations as well as the change in %. Time was taken after reading of the clinical report respectively the baseline report and arrangement of the images for the evaluation and stopped after the completion of the report.

### LMS software (Lesion Management Solutions, Median technologies, Valbonne Sophia Antipolis, France)

Each observer was previously introduced to LMS using five teaching cases. One data base was provided for each reader and baseline tumour measurements as well as the slice position of the target lesions were stored. Finally, an automatically generated quantitative report was created showing the patient and examination data and summarizes the measured values and sum LD. In follow-up reports, the calculated alteration of sum LD in % was provided additionally. Furthermore, snap shots of the target lesions were shown. Time was taken, after reading of the clinical report respectively the baseline report and arrangement of the images for the evaluation and stopped after printing of the report.

### Statistical analysis

The size of the target lesions (Diameter D) was recorded and the sum LD was calculated for each observer at baseline or follow-up, for both, PACS or LMS.

For the following calculations, the mean diameter (D_mean_) of identical lesions was calculated as reference, summarizing 1D measurements at baseline or follow-up from all readers and both software tools.

The accuracy of the 1D-measurements of the target lesions was quantified for each observer at baseline or follow-up for both, PACS or LMS, as [(*Δ D vs. D_mean_) / D_mean_] x 100 (%)*. The differences in measurements of the same lesions using PACS and LMS software were plotted against the mean value by using the Bland and Altman approach.

Intra-observer variability was assessed by comparing measurements of identical target lesions at baseline or follow-up, identified with both software tools for each observer as *[(Δ D_PACS_* *vs. D_LMS_)/D_mean_] x 100 (%)*

Accordingly, inter-observer variability was determined as the difference between measurements of identical target lesion for each pair of observers (O) at baseline or follow-up comparing same imaging systems (PACS vs. PACS resp. LMS vs. LMS) or different imaging systems (PACS vs. LMS resp. vice versa LMS vs. PACS) as *[(Δ D_OX_* *vs. D_OY_) / D_mean_] x 100 (%)*

To assess the overall response, the change of sum LD was calculated as *Δ sum LD = (sum LD_baseline_ - sum LD_follow-up_ / sum LD_baseline_) x 100 (%)*

Additionally, the summarized Δ sum LD was calculated per case, thus summarizing all evaluated target data (D_mean_) from both imaging systems and all observers per case.

The case evaluation time was calculated as mean for each and all observers at baseline or follow-up, for both, PACS and LMS.

Data are presented as mean, median, 10%, and 90% percentile. Measurements were compared with a paired two-tailed student’s t-test. Crosstabulation statistics were performed using the McNemar-Bowker Test. A p-value <0.05 was considered to indicate a statistical significance.

The study was carried out according to the Declaration of Helsinki.

## Results

A total of 320 RECIST reports were performed (4 observers x 20 cases x 2 evaluation time points x 2 software tools = 320).

As target lesions were not preselected, each observer identified independently up to five lesions per case. Five target lesions were selected in 44 cases, 4 target lesions in 22 cases, 3 target lesions in 39 cases, and 2 target lesions in 55 cases. No reports were completed with a single target lesion. The mean number of target lesion was 3.3 using PACS and 3.4 using LMS.

Altogether 120 different target lesions were defined. Twenty-one % of these target lesions have been selected consistently by all four readers and both software modalities. Twenty-nine % of the target lesions were selected only by one reader. A maximum of 10 different target lesions were observed in two patients with NSCLC and a carcinoma of the urothelium with multiple metastases to the liver, the lung and lymph nodes.

**FIGURES 1A, B:**
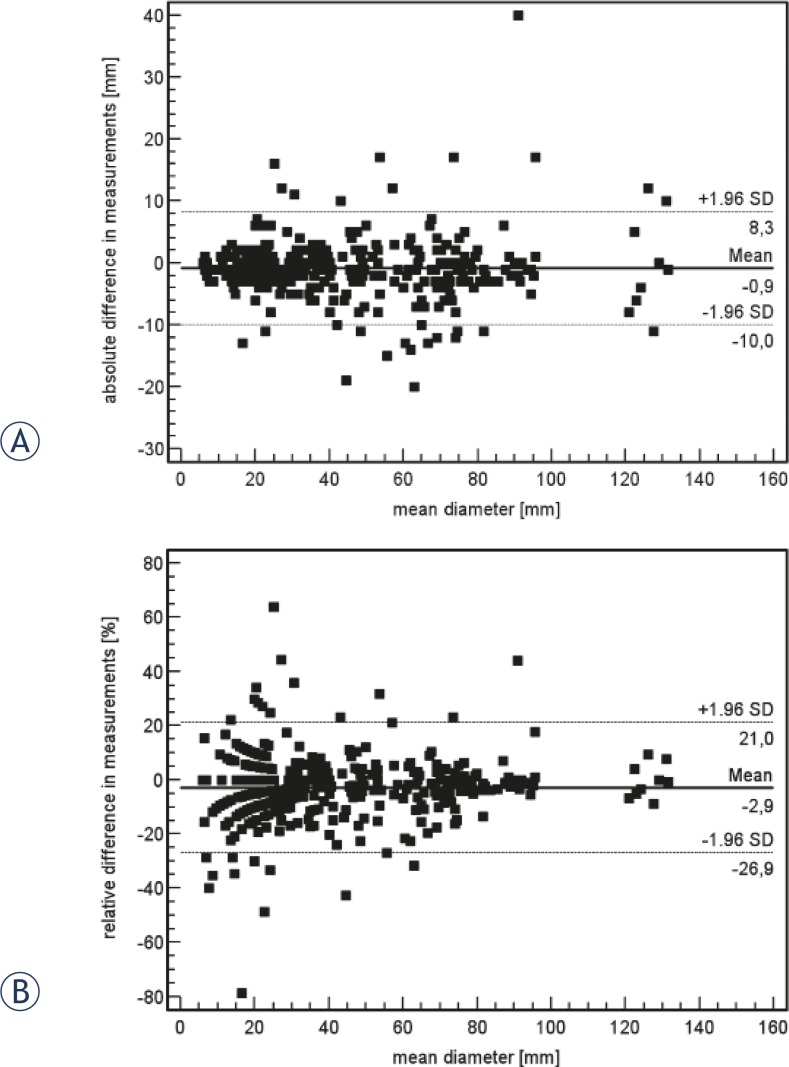
Graphs show the agreement of measurements of all lesions evaluated with PACS and LMS. Absolute (A) and relative (B) differences between both measurements are plotted against the mean diameter of the lesions. Mean difference is shown by a continuous line. Dashed lines indicate the limits of 1.96 standard deviations from the mean. A total of 93.8% (384 of 409) of the values lie within the 1.96 SDs of the mean (dashed lines).

Measurements of all lesions evaluated by PACS and LMS for baseline and follow-up assessment were compared. Figure 1 shows Bland-Altman analysis of the differences of percent diameter shrinkage measured by PACS and LMS compared to the average percent diameter stenosis by the two methods. The reproducibility of 1D measurements for all lesions was excellent with a mean difference in volume measurements amounted to −0.9 mm, with the 95% confidence interval ranging from −10 to 8.3 (Figure 1A). The mean relative difference amounted to −2.9 %, with a 95% confidence interval of −22.9 to 17.1 (Figure 1B).

Table 1 summarizes mean target size (mm) and variance. The smallest diameter of a target lesion was consistent to the RECIST guidelines 10 mm in baseline reports. The largest mean target diameter at baseline was 132 mm for a cohesive group of liver metastases. In follow-up examinations, the variance of target lesions ranged between 5 mm and 152 mm.

The mean sum LD (mm) and variance are presented in Table 2 showing comparable ranges.

The accuracy (%) of single 1D target measurements relatively to D_mean_ as well as the 10%- and 90%-percentile are documented in Table 3. A high mean accuracy of approximately 95% can be found.

The intra- and inter-observer variabilities for target measurements are displayed in Table 4, 5, and 6. The mean intra-observer variability was 5.0% at baseline and 6.8% at follow-up. The inter-observer variability was higher with values between 6.0–7.2% at baseline and 6.7–9.1% follow-up. Overall inter-observer variability was significantly higher than intra-observer variability for baseline and follow-up examinations (p<0.01 and p<0.05, respectively). There were no statistical significant differences comparing the both imaging systems, PACS and LMS.

**FIGURE 2:**
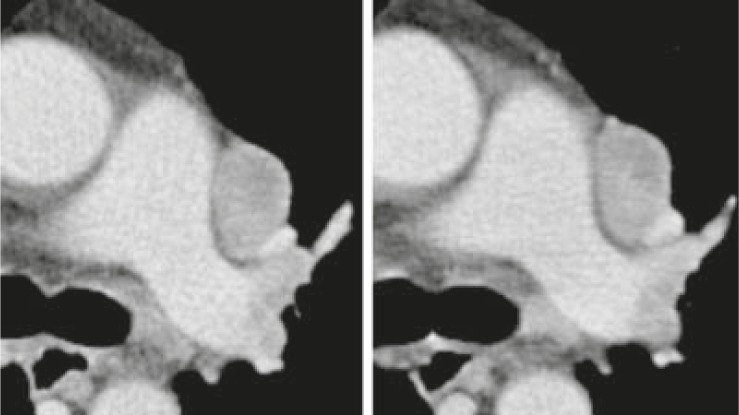
Tumor measurements of a well-marginated lymph node metastasis in a patient with renal cell carcinoma showed low mean inter-observer variabilities with 5.4 % for baseline (A) and 5.1% for follow-up (B), respectively. Mean intra-observer variability was low with 1.2% for (A) and (B).

**FIGURE 3:**
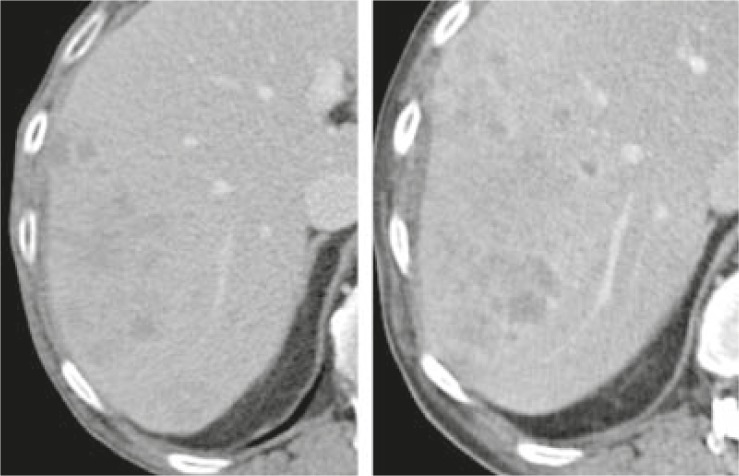
Poorly marginated, confluent liver lesions in a patient with NSCLC. Mean inter-observer variability was 14.9% for baseline (A) and 10.3% for follow-up (B), respectively. Mean intra-observer variability was 16.8% (A) and 7.7% (B).

Figures 2 and 3 illustrate variability of measurements in lesions with well-defined edges (Figure 2) and metastasis with irregular contours (Figure 3).

Table 7 lists the maximum and minimum Δ sum LD (%) and the overall response in all 20 cases. Despite a difference between maximum and minimum sum LD of 24%, misclassifications occurred in only 10 cases. There were no significant differences in response categorization for both imaging systems (p = 0.513). A high concordance could also be demonstrated to the summarized overall response, based on all assessed target lesions per case.

Table 8a–c shows the number of misclassifications for the overall response evaluation based on identical target lesions. Results for the assessment of the overall tumour response were compared for a single observer with all combinations of different observers (n=480) (a), a single observer vs. mean results of all observers (n=160) (b), and for different observers vs. mean results of all observers (n=480) (c). The number of misclassified cases can be reduced for the case assessment by a single observer and by mean results of all observers. Obviously, mean results of all observers equalize the outliers.

The mean time needed to prepare a baseline report was 286 s for PACS and 228 s for LMS software. At follow-up, mean time for PACS reporting was 267 s versus 196 s using LMS (Table 9). Thus, LMS induces a gain of time of 20.8% at baseline and 26.6% at follow-up (p<0.01).

## Discussion

In the study we assigned low intra- and inter-observer variability for target lesion measurements according to the RECIST 1.1 guidelines. However, the high variability in change of Δ sum LD shows the potential for misclassification of the overall response evaluation, but the reproducibility of RECIST reporting can be improved for the case assessment by a single observer and by mean results of multiple observers. Time required for the assessment and creation of a study report was decreased using custom software.

The assessment of tumour response in oncological clinical trials is usually based on serial measurements of primary tumour and metastases using CT examinations before and in the course of tumour therapy regimens. For consistent evaluation of tumour response concrete criteria for a standardized categorization of changes in tumour burden are necessary. 1D measurements for the calculation of tumour burden were introduced by Therasse *et al*.^4^ and the revised RECIST guidelines (version 1.1) were published in 2009 with the intention of further simplifying and standardizing tumour response criteria.^5^ Among others, the number of target lesions was restricted to a maximum of 5 with maximum of two lesions per organ. For target lesions, the longest diameter has to be assessed for tumour measurements except for lymph nodes, which are assessable as target lesion with a short axis > 15 mm. For quantifying tumour burden, the sum of longest diameter of all target lesions is calculated. Similarly, for some rare tumours, *i.e*. malignant mesothelioma, where the modified RECIST criteria were proposed, the tumour thicknesses are measured perpendicular to the chest wall in two sites at 3 levels and the sum of lesions’ diameters is calculated.^6^

In our study only target lesions were evaluated for reports of the tumour assessment in order to facilitate the comparison of the results of all four observers. Each observer individually defined target lesions out of the complete CT examination without any study-dependent pre-selection, so the setting of our study was closely adapted to clinical study reports.

A high intra- and inter-observer concordance of RECIST based quantifications of tumour burden is essential for a valid assessment of response to anticancer therapy regimens. Considering the agreement of measurements of identical lesions for each observer using PACS and LMS, intra-observer variability was low for all four observers with a mean difference of 5.9%. The inter-observer variability was slightly higher than the intra-observer variability with a mean variability of 7.1%. This is of special importance in case that different radiologists assess baseline and follow-up reports, as the RECIST guidelines do not advise for the same reader of tumour evaluation during an oncological trial.^5^ In contrary to our study, other studies evaluated the variability of tumour measurements using predefined single lesions. Erasmus *et al*. estimated mean intra- and inter-observer variability’s of 5.5% respectively 12.3% for 1D measurements, including irregular defined lesions.^7^ The lower discrepancies in our study might be due to a preferred selection of lesions with well-defined edges and avoiding of measurements of irregular shaped tumours’ lesions, as it is suggested for targets by the RECIST guidelines.

Despite the variability of single measurements the conclusive evaluation of the treatment response is of special interest for therapeutic decisions in clinical trials.^3^,^6^ According to RECIST guidelines, an increase of 20% of sum LD in follow-up examinations indicates disease progression (PD). A decrease of minimum 30% is considered as PR, whereas changes of sum LD between −30% and +20% is SD.^5^,^6^ In our study results of all observers showed excellent concordance for estimation of tumour response, but it has to be stated, that the mean difference of the Δ sum LD was 24%. Therefore, cases with tumour growth or tumour shrinkage in the region of the threshold for PD and PR will be problematic. In those cases standard deviation of single measurements may have an increased influence on the conclusion of the tumour response report. Furthermore, misclassification of overall response evaluation was higher if different observers assessed baseline and follow-up examinations, but can be reduced for the case assessment by a single reader and mean assessment of multiple readers.

A controversially discussed approach is the minimum number of target lesions needed for valid tumour evaluation.^8^–^10^ We confirmed a high accuracy of the treatment response categorization with up to five target lesions according to RECIST 1.1 compared to conclusive results summarizing all lesions. This summarized sum LD evaluation of all defined targets was closely to RECIST 1.0 criteria providing up to ten lesions for the tumour assessment. Darkeh *et al*. showed an increase of discrepancies in tumour response evaluation if less than four target lesions were defined for tumour measurements.^8^ In contrast, the evaluation of North Central Cancer Treatment Group trials determined two target lesions to be sufficient for concordant results. Also Zacharia *et al*. presented that the measurement only of one target lesion attained same classifications for tumour response in patients with colon cancer metastases to the liver.^10^

Simple 1D measurements of target lesions were equivalent using PACS or LMS. Thus, our study provides among others “repetitive” quantitative data. Nevertheless, the LMS software tool provides for the follow-up examinations the previous 1D target measurements, marked by a line and stored in the images. This is advantageous for serial measurements at follow-up reports, especially if different observers assess tumour burden during anticancer treatment. It would be interesting for further investigations, if inter-observer variability could be decreased by such a software tool in case that baseline and follow-up reports are performed by different readers. Considering the temporal effort required for the complete target evaluation and creation of a RECIST based report of tumour burden, there is a gain of time using LMS software, which might help to persuade radiologists to perform RECIST reports for each oncological patient.

A limitation of our study was a disproportionate incidence of the overall tumour response of “stable disease”. This is partly caused by the predetermination to assess only the development of target lesions, whereas non-target lesions and new lesions were not evaluated. It has been shown *e.g*. that in 60% of the cases PD is based on the occurrence of new tumourous lesions.^11^ Another explanation concerning PR was the fact, that baseline and first follow-up examinations of metastasized cancer patients were included to our study and PR may occur in the time course of the treatment. The potential saving of time using LMS could have been higher, as the readers are familiar with PACS for years, whereas the introduction of LMS based only on five teaching cases.

Perspectively, it will be of special interest to optimize the radiological evaluation of tumour burden and treatment response, with a special interest on new imaging techniques and further improvement of guidelines for tumour measurements.^12^,^13^ Future tumour response reports may provide volumetric tumour assessment and changes of tissue attenuation, leading to a more accurate and extended response evaluation. The volumetric measurement of pulmonary nodules is already feasible with numerous quantitative software tools and could be integrated into clinical routine.^14^,^15^ However, further increase of consistency of volumetric assessment of pulmonary nodules and low variability of semi-automated volume measurements will be required.^14^,^16^,^17^ For the complete tumour assessment semi-automated measurements of *e.g*. liver lesions and lymph nodes is necessitated and currently work in progress. Thus, up to now there are only a few results testing reproducibility and validity.^18^–^21^ Despite tumour shrinkage, a decrease of attenuation in contrast enhanced CT indicates tumour response, especially in the treatment with targeted therapies. Several studies declined an improvement of response evaluation after targeted therapy in *e.g*. metastatic renal cell carcinoma and squamous cell carcinoma of the upper aerodigestive tract when both, changes in tumour size and attenuation was assessed.^22^–^25^ Furthermore, Stacchiotti *et al*. demonstrated that additional evaluation of tumour attenuation increased predictive estimation of tumour response in patients with high-grade soft-tissue sarcomas.^26^

## Conclusions

We demonstrated in our clinical study low intra- and inter-observer variabilities for measurements of single target lesions, but the high variability in change of Δ sum LD reveals the potential for misclassification of the overall response according to the RECIST guidelines. Nevertheless, reproducibility of RECIST reporting can be improved for the case assessment by a single reader and mean results of multiple readers. Custom software shortened case-based evaluation time and further improvements might be challenging for therapy monitoring.

## Figures and Tables

**TABLE 1:** 1D measurement of target lesions (mm) for each observer using PACS or LMS at baseline or follow-up

**Evaluation**	**System**	**Observer**	**Mean target size (mm)**	**Minimum target size (mm)**	**Maximum target size (mm)**
Baseline	PACS	1	35.7	11	125
		2	40.9	10	132
		3	38.1	11	117
		4	38.8	12	121
	LMS	1	36.7	10	120
		2	41.7	11	126
		3	37.9	11	125
		4	41.1	11	126

Follow-up	PACS	1	34.9	6	129
		2	39.6	7	136
		3	37.3	6	152
		4	38.5	6	131
	LMS	1	35.0	6	129
		2	40.9	6	126
		3	35.5	5	133
		4	40.8	6	132

**TABLE 2:** Sum of the longest diameters of target lesions (mm) per case for each observer using PACS or LMS at baseline or follow-up

**Evaluation**	**System**	**Observer**	**Mean sum LD (mm)**	**Minimum sum LD (mm)**	**Maximum sum LD (mm)**
Baseline	PACS	1	118.3	35	261
		2	143.1	38	330
		3	120.1	34	312
		4	130.1	41	310
	LMS	1	127.4	29	296
		2	139.6	40	336
		3	130.6	39	305
		4	129.9	37	326

Follow-up	PACS	1	115.1	31	310
		2	136.5	34	359
		3	117.4	25	326
		4	129.0	33	342
	LMS	1	121.7	29	315
		2	136.9	36	356
		3	122.6	34	327
		4	128.4	28	359

**TABLE 3:** Accuracy of 1D measurements of target lesions in comparison to D_mean_ (%) for each observer using PACS or LMS at base-line or follow-up

**Evaluation**	**System**	**Observer**	**Median (%)**	**10% Percentile**	**90% Percentile**
Baseline	PACS	1	94.3	85.1	98.5
		2	94.8	88.5	99.7
		3	97.1	85.3	100
		4	95.8	84.4	99.6
		Mean	95.5		
	LMS	1	95.7	85.9	100
		2	95.3	84.8	99.9
		3	96.3	83.7	99.2
		4	95.7	84.6	98.9
		Mean	95.7		

Follow-up	PACS	1	93.9	79.2	99.4
		2	96.8	84.4	99.3
		3	96.6	84.1	100
		4	96.0	78.3	100
		Mean	95.8		
	LMS	1	96.0	79.7	99.6
		2	94.1	72.0	99.5
		3	94.5	78.5	99.3
		4	93.8	85.6	99.2
		Mean	94.6		

**TABLE 4:** Intra-observer variability for PACS vs. LMS at baseline or follow-up

**Evaluation**	**Observer**	**Median (%)**	**10% Percentile**	**90% Percentile**
Baseline	1	4.9	0.0	15.0
	2	4.9	0.0	14.1
	3	5.0	0.0	17.2
	4	5.4	0.0	17.8
	**Mean**	**5.0**		

Follow-up	1	5.2	0.0	18.9
	2	5.4	0.0	21.9
	3	6.9	0.9	29.2
	4	9.6	0.7	22.5
	**Mean**	**6.8**		

**TABLE 5:** Inter-observer variability. Difference of baseline 1D measurements of target lesions between two observers using PACS and/or LMS relative to mean tumour size (%)

**System**	**Observer pairs**	**Median (%)**	**10% Percentile**	**90% Percentile**
PACS vs. PACS	1/2	9.9	0	22.5
	1/3	5.6	0	21.6
	1/4	6.2	0	26.5
	2/3	6.8	0	15.9
	2/4	6.2	1.8	18.2
	3/4	4.7	0	22.3
	**Mean**	**6.5**		

LMS vs. LMS	1/2	7.6	1.5	20.6
	1/3	6.1	0	21.9
	1/4	5.6	2.1	23.2
	2/3	6.2	0	22.1
	2/4	5.8	0	22.6
	3/4	4.9	0	24
	**Mean**	**6.0**		

PACS vs. LMS	1/2	10.3	3	22.3
	1/3	8.4	0	21.9
	1/4	7.9	1.5	16.2
	2/3	5.7	0	23.2
	2/4	5.0	2.2	25.9
	3/4	6.2	0	22.3
	**Mean**	**7.2**		

LMS vs. PACS	1/2	6.6	0.7	12
	1/3	4.8	0	24
	1/4	5.8	0	25.3
	2/3	7.9	0	25.4
	2/4	6.2	0.3	25.7
	3/4	6.1	0	27.9
	**Mean**	**6.2**		

**TABLE 6:** Inter-observer variability. Difference of follow-up 1D measurement of target lesions between two observers using PACS and/or LMS relative to mean tumour size (%)

**System**	**Observer pairs**	**Median (%)**	**10% Percentile**	**90% Percentile**
PACS vs. PACS	1/2	9.3	0	26.3
	1/3	7.9	1.3	27.1
	1/4	8.0	1.2	33.2
	2/3	5.6	0	26.6
	2/4	4.3	0	18.8
	3/4	4.9	0	32.2
	**Mean**	**6.7**		

LMS vs. LMS	1/2	7.6	0.4	42.9
	1/3	6.9	0	30.7
	1/4	8.5	0	21.0
	2/3	7.6	0	42.9
	2/4	9.8	0	45.2
	3/4	9.1	1.6	30.5
	**Mean**	**8.2**		

PACS vs. LMS	1/2	10.8	1.9	41.5
	1/3	9.4	0	31.3
	1/4	11.4	2.4	28.6
	2/3	6.0	0	28.4
	2/4	7.8	1.1	17.1
	3/4	9.3	0	28.4
	**Mean**	**9.1**		

LMS vs. PACS	1/2	8.1	1.8	24.1
	1/3	6.5	2.4	27.0
	1/4	8.0	0.8	27.0
	2/3	5.9	0	45.2
	2/4	5.4	0	30.9
	3/4	8.4	0	38.5
	**Mean**	**7.1**		

**TABLE 7:** Tumour response per case (4 observers x 2 software tools x 20 cases = 160). Maximum and minimum sum LD (%), the difference (%), overall response, and the number of misclassifications are shown. Summarized Δ sum LD (%) and overall response were calculated based on D_mean_ of all target lesions per case, summarizing all observers and imaging systems. PR= Partial Response, SD= Stable Disease, PD= Progressive Disease; LD: sum of longest diameters

**Case**	**Maximum and minimum Δ sum LD (%)**		**Difference (%)**	**Overall Response**			**Misclassification**	**Summarized Δ sum LD (%)**	**Summarized overall response**
				**PR**	**SD**	**PD**			
1	−36	−13	23	2	6	0	2	−18	SD
2	−18	14	32	0	8	0	0	−4	SD
3	−17	14	31	0	8	0	0	−7	SD
4	0	23	23	0	6	2	2	14	SD
5	−27	−10	17	0	8	0	0	−22	SD
6	−10	5	15	0	8	0	0	2	SD
7	4	10	6	0	8	0	0	7	SD
8	−45	−36	9	8	0	0	0	−41	PR
9	−42	18	60	1	7	0	1	−11	SD
10	−5	15	20	0	8	0	0	7	SD
11	−14	−2	12	0	8	0	0	−7	SD
12	−14	20	34	0	8	0	0	11	SD
13	4	20	16	0	8	0	0	19	SD
14	−28	−16	12	0	8	0	0	−22	SD
15	−7	16	23	0	8	0	0	3	SD
16	0	10	10	0	8	0	0	6	SD
17	−27	−17	10	0	8	0	0	−22	SD
18	5	19	14	0	8	0	0	11	SD
19	−18	35	53	0	7	1	1	−3	SD
20	8	42	50	0	4	4	4	18	SD
**20**			**24**		**160**		**10**		

**TABLE 8:** Mean case evaluation time including reporting using PACS or LMS at baseline or follow-up

**Evaluation**	**Observer**	**Mean time (s)**		**p-value**
		**PACS**	**LMS**	
Baseline	1	395	310	<0.01
	2	232	212	<0.01
	3	298	175	<0.05
	4	219	216	<0.01
	**Mean**	**286**	**228**	**<0.01**

Follow-up	1	377	254	<0.01
	2	252	201	<0.01
	3	219	154	<0.01
	4	222	173	<0.01
	**Mean**	**267**	**196**	**<0.01**

## References

[1] Podobnik J, Kocijancic I, Kovac V, Sersa I (2010). 3T MRI in evaluation of asbestos-related thoracic diseases – preliminary results. Radiol Oncol.

[2] Inan N, Kilinc F, Sarisoy T, Gumustas S, Akansel G, Demirci A (2010). Diffusion weighted MR imaging in the differential diagnosis of haemangiomas and metastases of the liver. Radiol Oncol.

[3] Horvat M, Novakovic BJ (2010). Effect of response quality and line of treatment with rituximab on overall and disease-free survival of patients with B-cell lymphoma. Radiol Oncol.

[4] Therasse P, Arbuck SG, Eisenhauer EA, Wanders J, Kaplan RS, Rubinstein L (2000). New guidelines to evaluate the response to treatment in solid tumors. European Organization for Research and Treatment of Cancer, National Cancer Institute of the United States, National Cancer Institute of Canada. J Natl Cancer Inst.

[5] Eisenhauer EA, Therasse P, Bogaerts J, Schwartz LH, Sargent D, Ford R (2009). New response evaluation criteria in solid tumours: revised RECIST guideline (version 1.1). Eur J Cancer.

[6] Byrne MJ, Nowak AK (2004). Modified RECIST criteria for assessment of response in malignant pleural mesothelioma. Ann Oncol.

[7] Erasmus JJ, Gladish GW, Broemeling L, Sabloff BS, Truong MT, Herbst RS (2003). Interobserver and intraobserver variability in measurement of non-small-cell carcinoma lung lesions: implications for assessment of tumor response. J Clin Oncol.

[8] Darkeh MH, Suzuki C, Torkzad MR (2009). The minimum number of target lesions that need to be measured to be representative of the total number of target lesions (according to RECIST). Br J Radiol.

[9] Hillman SL, An MW, O’Connell MJ, Goldberg RM, Schaefer P, Buckner JC (2009). Evaluation of the optimal number of lesions needed for tumor evaluation using the response evaluation criteria in solid tumors: a north central cancer treatment group investigation. J Clin Oncol.

[10] Zacharia TT, Saini S, Halpern EF, Sumner JE (2006). CT of colon cancer metastases to the liver using modified RECIST criteria: determining the ideal number of target lesions to measure. AJR Am J Roentgenol.

[11] Therasse P, Le Cesne A, Van Glabbeke M, Verweij J, Judson I (2005). RECIST vs. WHO: prospective comparison of response criteria in an EORTC phase II clinical trial investigating ET-743 in advanced soft tissue sarcoma. Eur J Cancer.

[12] Curran SD, Muellner AU, Schwartz LH (2006). Imaging response assessment in oncology. Cancer Imaging.

[13] Shanbhogue AK, Karnad AB, Prasad SR (2010). Tumor response evaluation in oncology: current update. J Comput Assist Tomogr.

[14] Gavrielides MA, Kinnard LM, Myers KJ, Petrick N (2009). Noncalcified lung nodules: volumetric assessment with thoracic CT. Radiology.

[15] Creaney J, Francis RJ, Dick IM, Musk AW, Robinson BW, Byrne MJ (2011). Serum soluble mesothelin concentrations in malignant pleural mesothelioma: relationship to tumor volume, clinical stage and changes in tumor burden. Clin Cancer Res.

[16] Gietema HA, Schaefer-Prokop CM, Mali WP, Groenewegen G, Prokop M (2007). Pulmonary nodules: interscan variability of semiautomated volume measurements with multisection CT – influence of inspiration level, nodule size, and segmentation performance. Radiology.

[17] Wang Y, de Bock GH, van Klaveren RJ, van Ooyen P, Tukker W, Zhao Y (2010). Volumetric measurement of pulmonary nodules at low-dose chest CT: effect of reconstruction setting on measurement variability. Eur Radiol.

[18] De Vriendt G, Rigauts H, Meeus L (1998). A semi-automated program for volume measurement in focal hepatic lesions: a first clinical experience. J Belge Radiol.

[19] Heussel CP, Meier S, Wittelsberger S, Gotte H, Mildenberger P, Kauczor HU (2007). [Follow-up CT measurement of liver malignoma according to RECIST and WHO vs. volumetry]. [German]. Rofo.

[20] Keil S, Plumhans C, Behrendt FF, Stanzel S, Suehling M, Muhlenbruch G (2009). Automated measurement of lymph nodes: a phantom study. Eur Radiol.

[21] Keil S, Plumhans C, Behrendt FF, Stanzel S, Suehling M, Muhlenbruch G (2009). Semi-automated quantification of hepatic lesions in a phantom. Invest Radiol.

[22] Cowey CL, Fielding JR, Rathmell WK (2010). The loss of radiographic enhancement in primary renal cell carcinoma tumors following multitargeted receptor tyrosine kinase therapy is an additional indicator of response. Urology.

[23] Nathan PD, Vinayan A, Stott D, Juttla J, Goh V (2010). CT response assessment combining reduction in both size and arterial phase density correlates with time to progression in metastatic renal cancer patients treated with targeted therapies. Cancer Biol Ther.

[24] Smith AD, Lieber ML, Shah SN (2010). Assessing tumor response and detecting recurrence in metastatic renal cell carcinoma on targeted therapy: importance of size and attenuation on contrast-enhanced CT. AJR Am J Roentgenol.

[25] Petralia G, Preda L, Giugliano G, Jereczek-Fossa BA, Rocca A, D’Andrea G (2009). Perfusion computed tomography for monitoring induction chemotherapy in patients with squamous cell carcinoma of the upper aerodigestive tract: correlation between changes in tumor perfusion and tumor volume. J Comput Assist Tomogr.

[26] Stacchiotti S, Collini P, Messina A, Morosi C, Barisella M, Bertulli R (2009). High-grade soft-tissue sarcomas: tumor response assessment – pilot study to assess the correlation between radiologic and pathologic response by using RECIST and Choi criteria. Radiology.

